# Comparison of the genotypic and phenotypic properties of HIV-1 standard subtype B and subtype B/B′ *env* molecular clones derived from infections in China

**DOI:** 10.1038/s41426-018-0087-0

**Published:** 2018-05-16

**Authors:** Hui Xie, Jianhui Nie, Qingqing Chen, Weijin Huang, Youchun Wang

**Affiliations:** 1 Graduate School of Peking Union Medical College, No. 9 Dongdan Santiao, Dongcheng District, 100730 Beijing, China; 2 Division of HIV/AIDS and Sexually Transmitted Virus Vaccines, National Institutes for Food and Drug Control (NIFDC), No. 31 Huatuo Street, Daxing District, 102629 Beijing, China

## Abstract

Although a number of standardized human immunodeficiency virus 1 (HIV-1) pseudoviruses have been generated to assess neutralizing antibodies, subtype B/B′ has not been comprehensively characterized either genotypically or phenotypically. Full-length *env* genes were isolated from plasma samples derived from B/B′-infected individuals, including former plasma donors and men who had sex with men. The neutralization properties of these pseudoviruses were determined by testing against sCD4, 16 neutralizing monoclonal antibodies and 44 plasma samples, including samples from those infected with the three major prevalent clades in China. Twenty-eight pseudoviruses were successfully constructed, including 15 B′ and 13 B strains. Compared with subtype B strains from North America and Europe, the B′ strains from China showed greater variation in the V3 loop and higher susceptibility to the neutralizing antibody 2F5 and B/B′ plasma samples. The B′ strains from China showed significantly lower susceptibility to some trimer apex-binding neutralizing antibodies (PGT145, CH01, CH02, CH03, and CH04) than the B strains from Western countries. The 28 B-pseudotyped and B′-pseudotyped viruses were grouped into high, medium, and low clusters based on their overall neutralization sensitivity to plasma samples. The different genotypic and phenotypic properties of the standard subtype B from those of the Western viruses compared to the B and B′ strains from China suggest that clones from HIV-1-infected individuals in China are more suitable for the evaluation of candidate vaccines targeting the subtype B/B′ viruses circulating in China.

## Introduction

It is widely accepted that neutralizing antibodies (NAbs) play a key role in the efficacy of most currently used vaccines against viruses, such as those that cause smallpox and measles, polio, influenza, rabies, and human papillomavirus^1^. The protective potency of NAbs against human immunodeficiency virus 1 (HIV-1) has been confirmed in animal models^2–4^. However, no such antibody has been elicited by candidate vaccines in the participants of clinical trials, including RV114, which confers approximately 31% protection in low-risk heterosexual populations^5,6^. Recently, a significant achievement in the field of HIV-1 research has been the identification of a number of potent and broad-spectrum NAbs in naturally infected individuals^7–10^, which may provide a framework for the design of candidate vaccines to induce NAbs. The development of effective candidate HIV-1 vaccines depends not only on innovative immunogen design but also on standardized assays that can predict the protective effectiveness of these vaccines in vivo and guide the modification of immunogens.

Since the identification of HIV as the causative agent of acquired immunodeficiency syndrome (AIDS), a wide range of assays have been used to evaluate NAbs for the development of candidate vaccines, including T cell lines infected with T cell-line-adapted viruses, peripheral blood mononuclear cells infected with primary isolated viruses, and engineered cell lines infected with pseudoviruses or recombinant infectious viruses^11–18^. Of these methods, the pseudovirus-based neutralization assay (PBNA) using TZM-bl as the target cell is recommended as an optimized and validated approach for assaying serum samples in clinical vaccine trials^12^. A number of studies investigated the optimization, validation, international comparison, and technology transfer of this assay^11,16,19^. Pseudoviruses can be readily produced by cotransfecting mammalian 293T cells with *env*-expressing and *env*-defective molecular clones (expressing all HIV genes except *env*). One of the greatest advantages of a PBNA based on TZM-bl is the high versatility of the viral strains, which can be achieved simply by replacing the *env*-expressing plasmid with another plasmid in the transfection phase to generate a pseudovirus with the expected *env* protein on its surface.

Substantial efforts were directed to the diversification of pseudovirus pools to ensure that they are representative of the circulating viral strains targeted by candidate vaccines. Standardized panels of pseudoviruses are recommended to assess the potency and breadth of NAbs induced by candidate vaccines. A number of panels were constructed and standardized for this purpose, including HIV-1 clade B viruses, the most prevalent HIV strains in North America and Europe^20^, and clade C viruses, the most abundant subtype in Africa^21^. Based on the circulating strains in China, a large number of pseudoviral strains were generated and characterized, covering the main prevalent clades CRF01_AE, CRF07/08_BC, and B/B’^22–24^. Most genotypic and phenotypic studies of HIV in China focused on the two clades CRF01_AE^23^ and CRF07/08_BC^25^. Subtype B is one of the most widespread HIV-1 variants, accounting for approximately 11% of all infections worldwide. In addition to the pandemic B clade, four genetic variants have been described to date: B-Thai (B′), Trinidadian and Tobagian B, Korean B, and B″-GWGR. These variants represent well-established subclades of HIV-1 subtype B circulating in specific regions around the world^26^. In China, the B subtype is separated into two distinct variants: the pandemic B subtype and the B′ type^27^. In the 1990s, almost 50% of all HIV-1 infections were attributed to the B′ strains, most of which were detected in former plasma donors (FPDs). With the implementation of stringent monitoring and disposable blood collection materials, the prevalence of B′ strains decreased dramatically to <10% in 2006^28^. However, B′ strains were recently reported to have spread from FPDs to the general population by sexual transmission^29^. In China, subtype B strains account for approximately 28.25% of infections in the population of men who have sex with men (MSM), which is the most vulnerable population because of its high-risk sexual behaviors. Genotypic and phenotypic differences were identified between subtype C and CRF07/08_BC strains isolated in China, suggesting that pseudoviral strains derived from the same target region are most suitable for the evaluation of a candidate vaccine^25^.

In this study, we constructed a pool of subtype B and B′ *env*-pseudotyped viruses and comprehensively investigated their genotypic and phenotypic characteristics. A systematic analysis was conducted to compare the differences between the pseudoviruses derived from strains B′, B isolated in China, and B isolated in North America and Europe.

## Results

### Genetic characterization of molecularly cloned full-length *env* genes

**Fig. 1:**
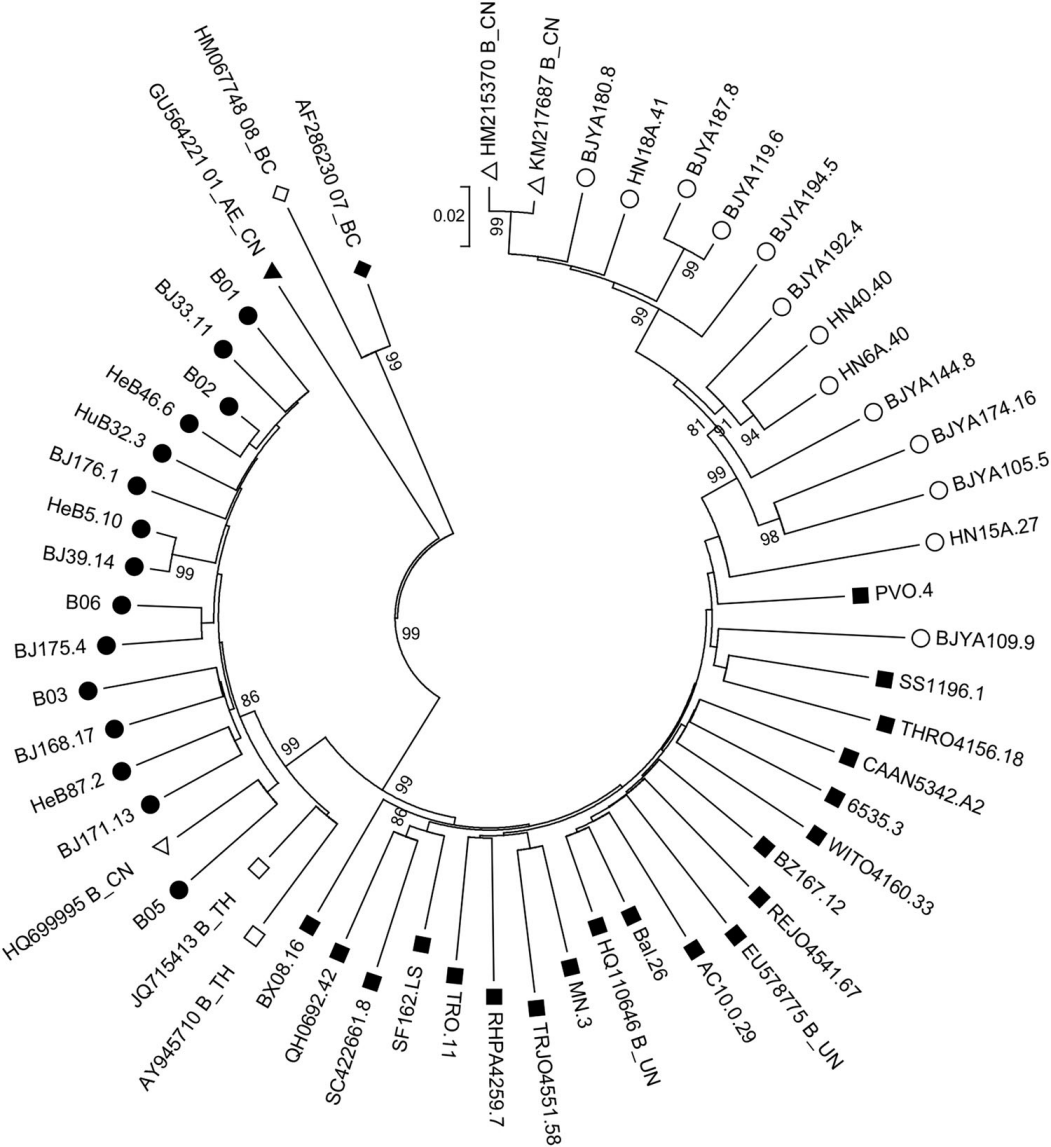
Phylogenetic tree of gp160 sequences derived from HIV-1 B/B′ infections and other subtype reference strains. Hollow circles (○) represent B strains from China; solid circles (●) represent B′ strains from China; hollow squares (□) represent reference B strains from Thailand; solid squares (■) represent reference B strains from Europe and America; hollow triangles (△) represent reference B strains from China; solid triangles (▲) represent subtype 01_AE_CN strains from China; hollow diamonds (◊) represent reference 08BC strains from China; and solid diamonds (♦) represent reference 07BC strains from China. The numbers on the nodes are bootstrap values, which indicate branch support on the phylogenetic tree. Only bootstrap values >80% are shown

Twenty-eight HIV-1 B/B′ molecular *env* clones, of which 19 were derived from FPDs and nine were derived from an MSM population, were constructed (Table 1). To compare the differences in B/B′ isolates in China and the B strains prevalent in North America and Europe, six tier 1 and 12 tier 2 molecularly cloned *env*-expressing plasmids^20^ (designated B_EU) were used in a comparative analysis, and pandemic B and B′ strains isolated from Chinese individuals were designated B_CN and B′_CN, respectively. A phylogenetic analysis of full-length gp160 nucleotide sequences demonstrated that a wide spectrum of genetic diversity was present among most clones, as is expected for independent infections. Of the 28 isolates originating from China, 15 were grouped as clade B′, all of which were derived from FPDs. All nine clones from MSM and the remaining four from FPDs clustered as subtype B (Fig. 1). The B_EU, B_CN, and B′_CN strains clustered on relatively separated branches with the corresponding reference strains, clearly indicating the distinct genotypic differences between the three pools of strains.

**Table 1:** Basic information and characterization of 28 HIV-1 B/B′ clones

Clone	Transmission	Location	Top 4 V3 peptides	Subtype
HeB5.10	Plasma donor	Hebei, China	GPGQ	B′
HuB32.3	Plasma donor	Hubei, China	GPGK	B′
BJ33.11	Plasma donor	Beijing, China	GQGR	B′
BJ39.14	Plasma donor	Beijing, China	GPGQ	B′
HeB87.2	Plasma donor	Hebei, China	GPGR	B′
BJ168.17	Plasma donor	Beijing, China	GPGQ	B′
BJ171.13	Plasma donor	Beijing, China	GQGR	B′
BJ176.1	Plasma donor	Beijing, China	GPGK	B′
BJ175.4	Plasma donor	Beijing, China	GPGK	B′
HeB46.6	Plasma donor	Hebei, China	GLGR	B′
B02^a^	Plasma donor	Gansu, China	GPGQ	B′
B05 ^a^	Plasma donor	Hebei, China	GPGR	B′
B06 ^a^	Plasma donor	Beijing, China	GPGK	B′
B01 ^a^	Plasma donor	Hebei, China	GPGQ	B′
B03 ^a^	Plasma donor	Hebei, China	GPGR	B′
HN6A.40	Plasma donor	Henan, China	GLGR	B
HN15A.27	Plasma donor	Henan, China	GPGG	B
HN18A.41	Plasma donor	Henan, China	AGGR	B
HN40.40	Plasma donor	Henan, China	GWGR	B
BJ105.5	MSM	Beijing, China	GLGR	B
BJ109.9	MSM	Beijing, China	GPGR	B
BJ119.6	MSM	Beijing, China	AGGG	B
BJ144.8	MSM	Beijing, China	GWGR	B
BJ174.16	MSM	Beijing, China	GWGR	B
BJ180.8	MSM	Beijing, China	VGGR	B
BJ187.8	MSM	Beijing, China	AGGG	B
BJ192.4	MSM	Beijing, China	GWGR	B
BJ194.5	MSM	Beijing, China	SPGR	B

The letter 'a' indicates that the clone was derived from a previous study^22^

**Fig. 2:**
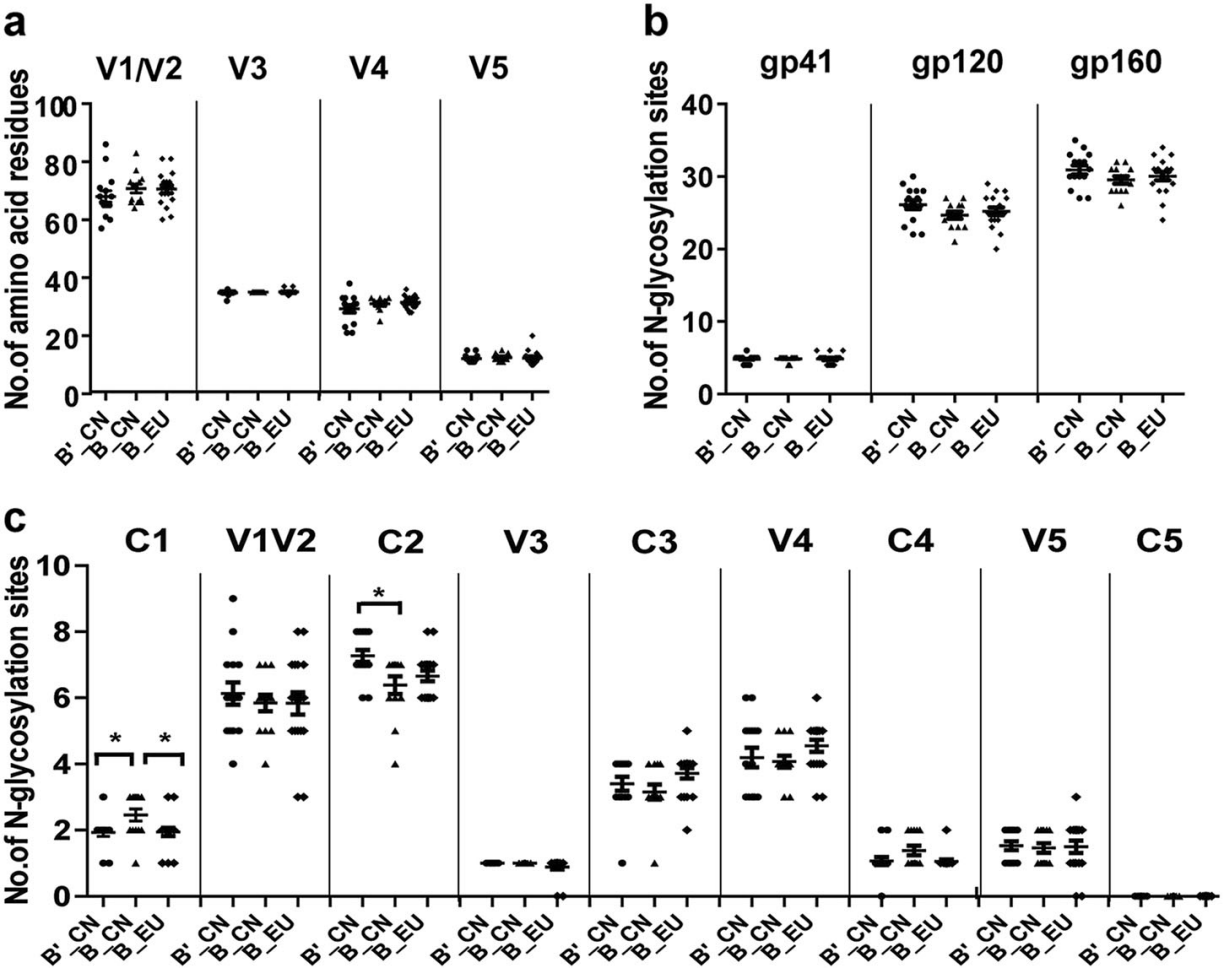
Comparison of the number of amino acid residues in the variable loops (**a**) and the number of potential N-linked glycosylation sites (**b**, **c**) in the subtype B′_CN (*n* = 15), B_CN (*n* = 13), and B_EU (*n* = 18) *env* sequences

The natural variation in the structure of the *env* trimeric spikes on the HIV-1 virion is predicted to influence the neutralization phenotype of the virus. In particular, epitope exposure may be partially influenced by the size and structure of the variable loops and the positions of N-linked glycosylation sites^20,22,23^. When the number of amino acids in the different loops were analyzed, no significant differences were observed in the lengths of the V1/V2, V3, V4, and V5 regions among these groups (B′_CN, B_CN, and B_EU) (Fig. 2a). The number of potential N-linked glycosylation sites (PNLGs) in the 28 HIV-1 B/B′ clones and subtype B_EU clones were also compared. No significant variation was found in the number of PNLGs in gp160, gp120, or gp41 (Fig. 2b). When the PNLG analysis focused on the conserved and variable regions, significant differences were observed in the PNLG numbers in C1 and C2 and between B′_CN and B_CN (one-way analysis of variance (ANOVA), *p* < 0.05; Fig. 2c).

The V3 loop of HIV-1 gp120 is associated with HIV-1 entry into the host cell^30^ and its pathogenicity^31,32^, and the four amino acid residues at the tip of the V3 loop play a key role. In this study, most of the tetramers of the V3 loop in subtype B_EU were GPGR (88.9%), among which only strains REJO4541.67 and THRO4156.18 contained APGR and GPGG, respectively. Compared with the tetramers of the B_EU isolates, those of the B/B′ strains cloned from Chinese infected individuals showed greater variation. The 15 B′_CN clones presented five types of tetrads: GPGQ (33.3%), GPGK (26.7%), GPGR (20%), GQGR (13.3%), and GLGR (6.7%). Even greater variation at the four amino acid residues at the tip of the V3 loop was observed in the B_CN strains, with eight types being identified: GWGR, GLGR, SPGR, VGGR, AGGR, GPGG, GPGR, and AGGG (Table 1).

### *env*-pseudotyped virus susceptibility to bnmAbs

**Fig. 3:**
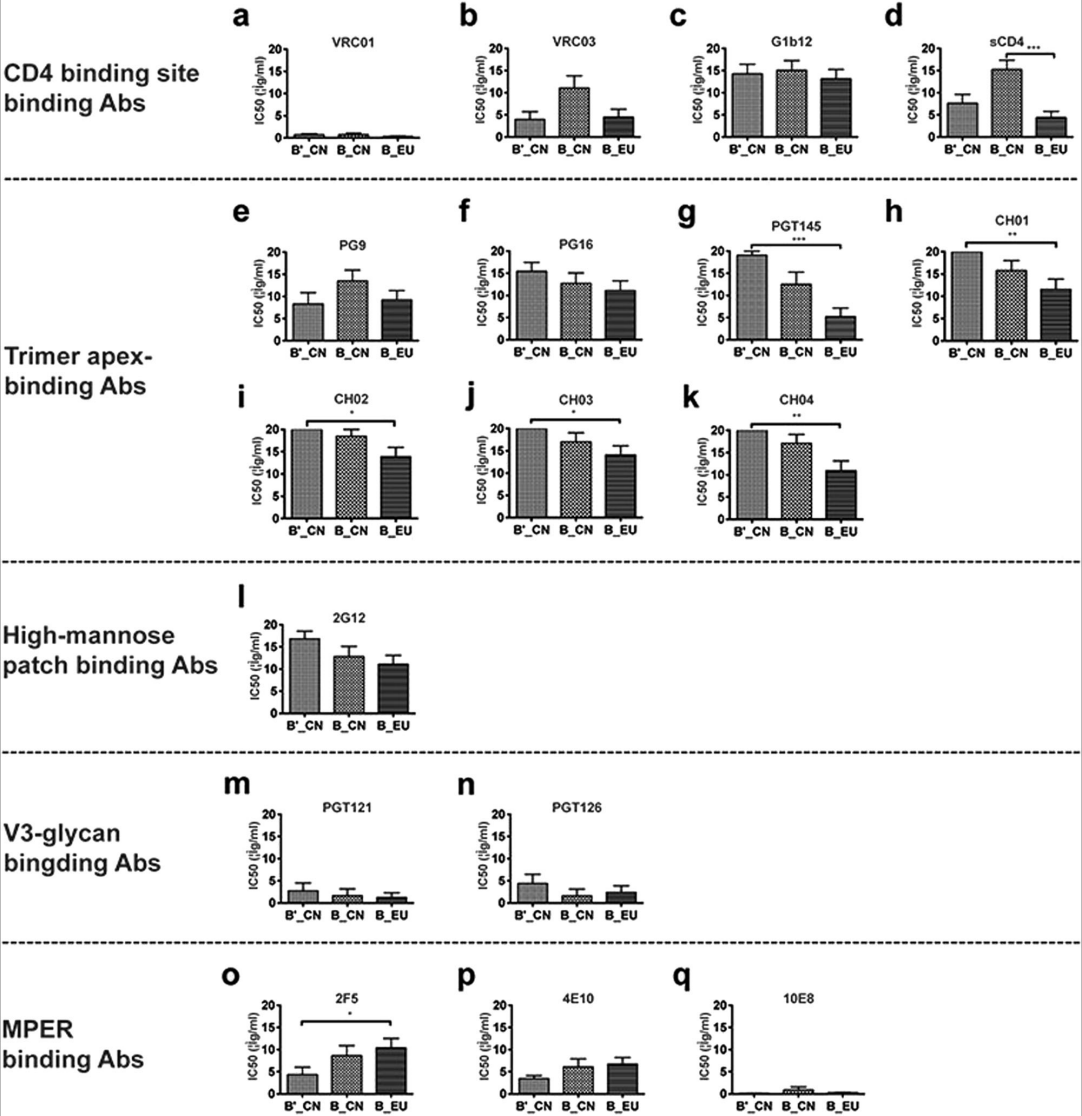
Comparison of the neutralizing phenotypes of sCD4 and 16 broadly neutralizing monoclonal antibodies (bnmAbs) against 46 *env* clones (**a**–**q**). *p < 0.05, **p < 0.01, and ***p < 0.0001

Neutralization phenotypes were investigated by testing the pseudoviruses against sCD4 and 16 broadly neutralizing monoclonal antibodies (bnmAbs), which were divided into five classes based on their binding sites (Table 2 and Fig. 3). Of the antibodies targeting the CD4-binding site, VRC01 showed the greatest neutralizing potency. All 28 newly constructed B′_CN and B_CN pseudoviruses together with the 18 B_EU strains were efficiently neutralized by VRC01, with half-maximal inhibitory concentration (IC_50_) values <10 μg/ml. VRC03 showed high neutralizing potency when tested against the B′_CN (13/15) and B_EU (15/18) strains. By contrast, only 6 of 13 B_CN strains were efficiently inhibited by VRC03. Most pseudoviruses from the three groups were resistant to mAb b12. No significant difference was observed among the three groups of pseudoviruses in their susceptibility to the CD4-binding site bnmAbs (Fig. 3a–c). Significant differences were observed when sCD4 was tested against the B_CN and B_EU strains (Fig. 3d). The main differences were attributable to the B_CN strains derived from MSM individuals (designated BJYAxx), all of which showed a resistant phenotype to sCD4. Although the three bnmAbs and sCD4 all target the CD4-binding site, they showed different potencies and breadth, potentially due to subtle differences in their epitopes.

**Table 2:** Neutralization susceptibility of the pseudoviruses to sCD4 and bnmAbs

Class of antibody	CD4-binding site-binding Abs				Trimer apex-binding Abs								High-mannose patch-binding Abs	V3-glycan-binding Abs		MPER-binding Abs		
	Clone ID	sCD4	G1b12	VRC01	VRC03	PG9	PG16	PGT145	CH01	CH02	CH03	CH04	2G12	PGT121	PGT126	2F5	4E10	10E8
B′_CN	HeB5.10	>10	3.99	1.45	4.62	0.75	6.26	>10	>10	>10	>10	>10	>10	0.02	0.01	>10	9.81	0.09
	HuB32.3	1.67	>10	0.27	0.08	>10	>10	>10	>10	>10	>10	>10	3.27	0.08	0.02	0.68	1.00	0.22
	BJ33.11	4.56	>10	0.17	7.77	0.76	>10	>10	>10	>10	>10	>10	>10	0.07	0.04	2.81	7.40	0.14
	BJ39.14	>10	3.09	0.81	>10	1.72	>10	>10	>10	>10	>10	>10	>10	0.02	0.01	3.22	4.78	0.01
	HeB87.2	>10	>10	0.21	0.15	>10	>10	>10	>10	>10	>10	>10	>10	0.16	0.01	3.92	3.90	0.20
	BJ168.17	4.16	>10	3.79	4.41	>10	>10	>10	>10	>10	>10	>10	>10	>10	>10	0.82	1.22	0.09
	BJ171.13	>10	2.41	0.72	0.83	>10	>10	6.19	>10	>10	>10	>10	>10	0.01	0.01	4.69	5.46	0.19
	BJ175.4	1.87	>10	0.12	0.08	0.21	3.12	>10	>10	>10	>10	>10	>10	>10	>10	0.92	1.54	0.04
	BJ176.1	1.31	>10	0.05	0.63	0.28	>10	>10	>10	>10	>10	>10	>10	0.19	>10	1.02	2.01	0.06
	HeB46.6	2.13	>10	0.02	0.04	0.27	0.82	>10	>10	>10	>10	>10	>10	0.01	0.01	0.36	0.69	0.04
	B01	1.44	>10	1.25	>10	>10	>10	>10	>10	>10	>10	>10	>10	0.12	3.03	0.54	1.28	0.01
	B03	3.80	4.16	0.92	0.38	0.28	1.27	>10	>10	>10	>10	>10	7.69	0.01	0.01	2.63	2.98	0.14
	B06	4.35	>10	0.17	0.24	0.21	>10	>10	>10	>10	>10	>10	>10	0.01	2.50	3.02	4.67	0.44
	B02	1.07	0.25	0.06	0.08	0.22	>10	>10	>10	>10	>10	>10	1.18	0.02	0.03	0.45	0.84	0.02
	B05	7.61	>10	0.01	0.01	>10	>10	>10	>10	>10	>10	>10	>10	0.04	0.04	>10	3.68	0.28
B_CN	HN6A.40	>10	3.86	1.29	>10	6.87	9.93	0.13	>10	>10	>10	>10	>10	0.35	0.03	5.79	7.35	0.21
	HN18A.41	2.74	>10	0.03	0.03	5.78	5.86	0.03	0.74	>10	1.21	1.97	>10	0.04	0.04	3.18	4.87	0.18
	HN15A.27	2.06	7.88	0.12	0.66	0.98	>10	>10	>10	>10	>10	>10	>10	>10	>10	2.48	2.50	0.03
	HN40.40	4.33	0.50	0.03	>10	>10	>10	>10	>10	>10	>10	>10	2.14	0.02	0.04	0.58	0.78	0.03
	BJYA105.5	>10	>10	0.49	>10	>10	2.11	>10	>10	>10	>10	>10	6.92	0.31	0.01	8.64	5.80	0.02
	BJYA109.9	>10	>10	0.06	0.01	0.20	0.01	0.03	0.07	0.06	0.08	0.05	8.58	0.01	0.02	2.44	3.34	0.24
	BJYA119.6	>10	>10	0.05	1.98	>10	>10	>10	>10	>10	>10	>10	>10	0.04	0.03	3.83	2.80	0.24
	BJYA144.8	>10	>10	0.25	0.30	1.91	3.87	>10	>10	>10	>10	>10	4.15	0.01	0.01	4.38	5.27	0.32
	BJYA174.16	>10	>10	0.13	>10	>10	>10	>10	>10	>10	>10	>10	4.71	0.01	0.01	>10	0.41	0.12
	BJYA180.8	>10	>10	1.81	>10	>10	3.19	0.10	>10	>10	>10	>10	>10	0.11	0.17	>10	>10	9.17
	BJYA187.8	>10	>10	0.05	>10	>10	>10	>10	>10	>10	>10	>10	>10	0.01	0.01	>10	>10	0.54
	BJYA192.4	>10	3.51	0.53	0.51	>10	>10	>10	>10	>10	>10	>10	0.11	0.08	0.02	0.90	1.44	0.07
	BJYA194.5	8.89	>10	4.26	>10	>10	>10	2.15	4.16	>10	>10	>10	>10	0.06	0.02	>10	4.66	0.31
B_EU	6535.3	0.82	>10	1.76	1.21	1.87	>10	>10	0.39	>10	>10	0.41	6.18	0.00	0.02	4.48	2.97	0.07
	QH0692.42	0.50	2.34	0.61	0.24	>10	>10	>10	>10	>10	>10	>10	7.34	0.25	0.03	2.07	3.81	0.07
	PVO.4	3.51	>10	0.29	0.24	>10	>10	0.10	>10	1.85	1.84	2.84	4.80	0.10	0.02	>10	>10	0.64
	TRO.11	7.72	>10	0.30	0.16	>10	2.16	0.04	>10	>10	>10	>10	0.66	0.01	0.05	>10	4.69	0.03
	AC10.0.29	3.99	>10	0.81	>10	0.55	0.04	0.01	0.45	1.14	3.80	0.14	>10	0.03	0.22	1.12	1.18	0.04
	WITO4160.33	2.46	>10	0.11	>10	0.02	0.01	0.003	0.01	0.01	>10	0.01	1.89	0.60	1.95	1.50	2.03	0.07
	TRJO4551.58	>10	>10	0.05	0.01	3.04	2.70	>10	>10	>10	>10	>10	>10	0.51	0.04	>10	4.87	0.16
	REJO4541.67	1.82	>10	0.05	0.05	0.10	1.71	0.002	0.08	0.12	0.24	>10	>10	0.23	>10	1.41	2.38	0.11
	RHPA4259.7	0.74	0.74	0.03	4.33	>10	3.74	0.05	1.26	>10	2.41	3.42	>10	0.02	0.03	>10	>10	0.24
	THRO4156.18	0.96	5.96	0.03	5.21	>10	7.20	0.04	>10	4.16	>10	2.33	7.20	0.02	0.01	>10	4.07	0.29
	CAAN5342.A2	6.78	>10	0.62	8.98	>10	>10	3.27	>10	>10	>10	>10	>10	0.02	0.19	>10	>10	1.60
	SC422661.8	3.65	2.83	0.05	0.02	4.88	>10	0.02	>10	>10	>10	>10	4.62	0.05	0.06	2.33	3.77	0.11
	BaL.26	0.10	0.48	0.03	>10	0.72	>10	2.57	>10	>10	>10	>10	2.80	0.02	0.08	3.60	6.10	0.31
	BX08.16	0.25	>10	0.10	0.05	0.78	>10	0.01	0.53	1.66	0.52	1.09	>10	0.01	0.01	4.27	3.61	0.14
	BZ167.12	>10	>10	0.45	0.11	5.58	>10	0.39	>10	>10	>10	>10	2.30	>10	>10	1.19	1.75	0.35
	MN.3	0.91	3.33	0.17	0.03	5.12	1.92	6.21	2.77	>10	4.32	4.05	>10	0.01	0.01	>10	1.93	0.25
	SS1196.1	4.30	>10	0.13	0.02	2.71	0.28	0.88	2.23	>10	>10	1.94	>10	0.02	0.01	>10	9.10	0.10
	SF162.LS	0.09	0.38	0.03	0.02	>10	>10	>10	>10	>10	>10	>10	1.58	0.01	0.01	5.31	7.86	0.04

The IC_50_ value of bnmAbs is defined as the concentration (μg/ml) that causes a 50% reduction in the RLUs compared with the virus control wells after background (RLU) subtraction

*IC*_*50*_ half-maximal inhibitory concentration, *bnmAbs* broadly neutralizing monoclonal antibodies, *MPER* membrane proximal external region, *RLU* relative luminescence unit

Among the second class of antibodies targeting the trimeric apex, PG9 performed better when tested against the B′_CN pseudoviruses than against B_CN or B_EU (Fig. 3e). PG16 showed the opposite tendency, with greater neutralization efficiency against the subtype B strains (Fig. 3f). N160 is a critical site for both PG9 and PG16^33^. PG9 and PG16 bind to the V1 and V2 variable loops of gp120^8^. Three pseudoviruses with a mutation at N160 (B01: N160D, QH0692.42: N160S, and SF162.LS: N160K) all showed a resistant phenotype for both NAbs. PGT145 (Fig. 3g) and CH01-04 (Fig. 3h–k) showed similar neutralizing activities, performing best against B_EU strains and worst against B′_CN strains. Significant differences were observed between these two groups of pseudoviruses.

The glycan-dependent mAb 2G12 recognizes a conformational epitope involving N295, N332, N339, N386, and N392^34^. Among these N-linked glycans, N295 and N332 play key roles in the attachment of 2G12 to gp120^24^. In this study, viruses (B06: N295I, HN18A.41: N295E, BJYA119.6: N295K, BJYA187.8: N295K, BJYA194.5: N295S, AC10.0.29: N295K, and CAAN5342.A2: N295T) with a mutation at N295 all showed a resistant phenotype. When the three groups of pseudoviruses were compared, no significant differences were detected. Only three of the 15 B′_CN pseudoviruses were neutralized by 2G12, a glycan-dependent mAb, at a concentration <10 μg/ml. Twenty-seven of 46 pseudoviruses presented a resistant phenotype at a concentration of 10 μg/ml (Fig. 3l).

For the V3-glycan-binding mAbs, the newly isolated bnmAbs (PGT121 and PGT126) showed high neutralizing potency against these pseudoviruses. Only four pseudoviruses (two from B′_CN, one from B_CN and one from B_EU) were not neutralized at a PGT121 concentration of 10 μg/ml. Similar neutralization potencies were observed for PGT126, which could neutralize almost all pseudoviruses except six (three from B′_CN, one from B_CN and two from B_EU) (Fig. 3m, n).

Among the MPER-binding Abs (Fig. 3o–q), 10E8 showed the greatest neutralizing potency and breadth, and 4E10 also showed high inhibition efficiency. Only five pseudoviruses (two from B_CN and three from B_EU) were not neutralized by 10E8 at a concentration of 10 μg/ml. Infection by all 15 B′_CN strains was completely inhibited by 4E10, whereas 86.7% of B′_CN, 69.2% of B_CN, and 55.6% of B_EU viruses were neutralized by 2F5. The susceptibility of B′_CN and B_CN differed significantly. The 2F5 antibody binds to conserved linear epitopes and recognizes the epitope formed by the peptide sequence ELDKWA, in which LDKW is critical, particularly L665^35^. In this study, pseudoviruses of the B′_CN strains were more sensitive to 2F5 than the subtype B_EU viruses, which may have been due to the conservation in 662E, 665K, and 667A.

### Susceptibility of *env*-pseudotyped viruses to HIV-1-positive plasma

**Fig. 4:**
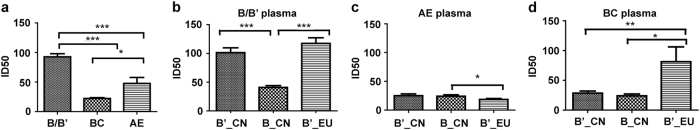
Comparison of the neutralizing activities of HIV-1 B/B′, CRF07/08_BC, and CRF01_AE plasmas against 46 HIV-1 pseudoviruses (*p < 0.05, ***p < 0.0001) (**a**). Neutralizing sensitivities of HIV-1 B/B′ and subtype B_EU pseudoviruses compared with those of the subtype BB′, CRF07/08_BC, and CRF01_AE plasma panels (**b**–**d**). *p < 0.05, **p < 0.001, and ***p < 0.0001

To further characterize the neutralization properties of the B/B′ pseudoviruses, 44 plasma samples (24 B/B′, 10 CRF01_AE, and 10 CRF07/08_BC) were used to test 46 pseudoviruses. The neutralization efficiency of the plasma samples showed a type-specific pattern with a neutralization potency in the order subtype B/B′ > subtype AE > subtype BC (Kruskal–Wallis test; Fig. 4a).

When the pseudoviruses were compared by testing them against B/B′ plasma samples, B′_CN was more susceptible than B_CN or B_EU (*p* < 0.0001). The B′_CN and B_CN pseudoviruses showed similar sensitivities to the CRF01_AE plasma samples, but those of B_CN and B_EU differed significantly (*p* < 0.05). No significant difference was observed in the susceptibility of B′_CN and B_CN to CRF07/08_BC plasma, whereas B_EU was more susceptible than either B′_CN or B_CN (Kruskal–Wallis test; Fig. 4b–d).

**Fig. 5:**
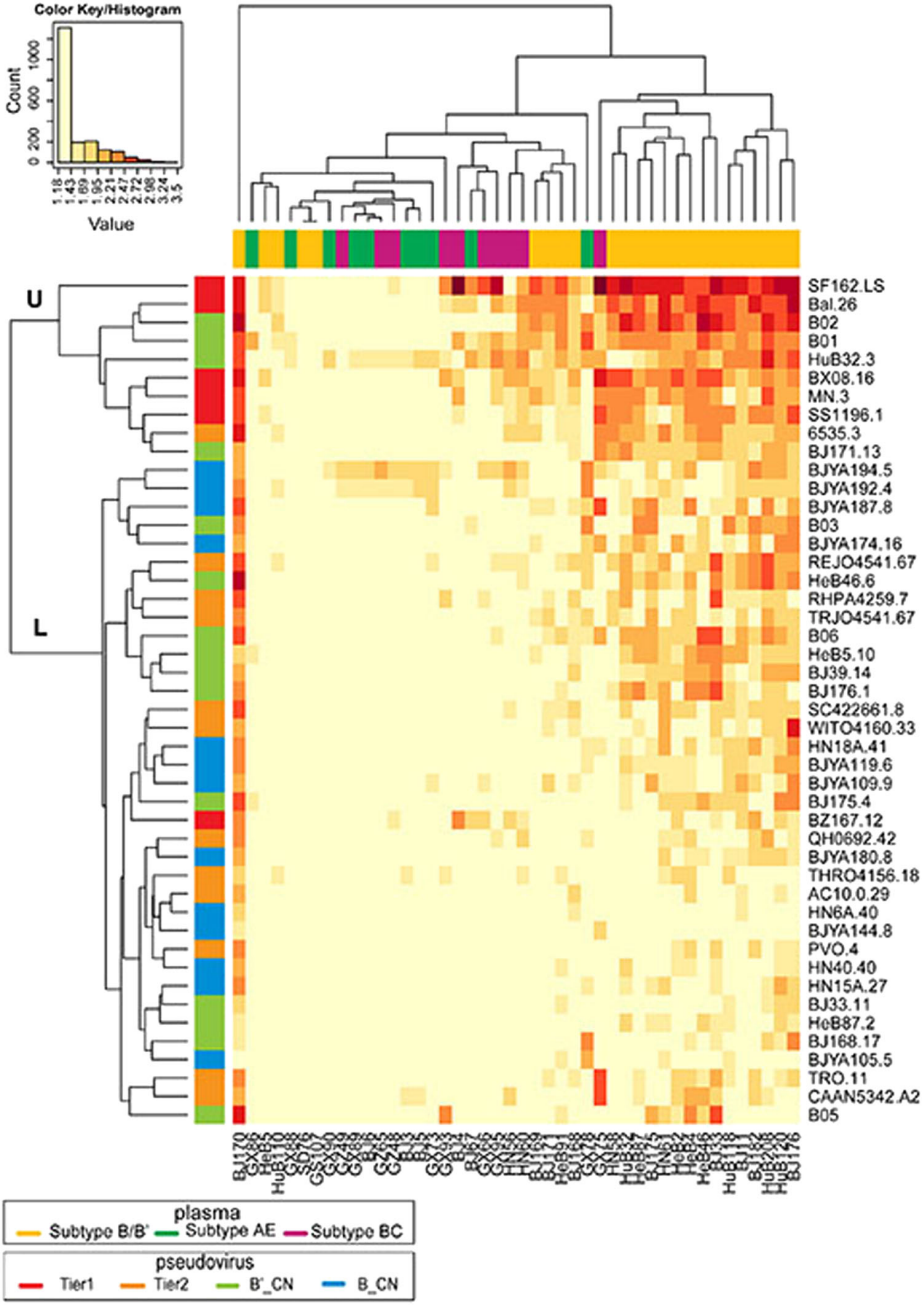
**Heatmap to analyze the relationship of neutralization sensitivity between the plasma panel and pseudovirus panel**

To investigate the susceptibility of the pseudoviruses and the neutralization potency of the plasma samples systematically, a heatmap was constructed to summarize 2024 neutralization tests. According to their neutralization patterns, the 46 pseudoviruses were divided into two main clusters: the upper cluster (U) and lower cluster (L). Among the tier 1 B_EU pseudoviruses, only BZ167.12, located in the L cluster, showed low sensitivity to the 46 plasmas. Of all tier 2 B_EU pseudoviruses, only strain 6535.3 in the U cluster, which was most sensitive to neutralization, showed a higher sensitivity than the tier 1 viruses identified previously. Four B′_CN viruses were grouped into the U cluster and defined as tier 1 viruses (B02, B01, HuB32.3, and BJ171.13). The remaining 24 strains of B/B′ viruses isolated in China and the tier 2 B_EU viruses were located in the L cluster and defined as tier 2 viruses (Fig. 5).

## Discussion

A major challenge in developing effective vaccines against HIV-1 is the great genetic and antigenic variability of the virus, which are also significant obstacles to assess the immunogenicity of candidate vaccines. The development and standardization of a PBNA based on TZM-bl has partially resolved this problem. The critical element of this assay is the pseudovirus used, which should be representative of the viruses circulating in the vaccine-targeting region. The epidemic of HIV-1 in China has a unique pattern, as CRF01_AE, CRF07/08_BC, and B/B′ comprise more than 90% of the reported cases. Standardized pseudovirus panels for CRF01_AE^23^ and CRF07/08_BC^16,25^ were constructed and used in the analysis of vaccine-induced NAbs. A standardized pseudovirus panel for the third prevalent clade, B/B′, is urgently required. Here, we investigated the genotypic and phenotypic characteristics of 28 HIV-1 B/B′ strains derived from FPDs and MSMs in China and compared them with standard B_EU clones.

An important goal of our study was to determine the subtype that is likely to be the best immunogen for a candidate vaccine and to evaluate its efficacy as a NAb-based vaccine for use in China. To this end, we compared the genetic and phenotypic properties of Env pseudoviruses derived from *env* sequences isolated from HIV-1 infections in China with those of standard reference pseudoviruses. A phylogenetic analysis of full-length gp160 nucleotide sequences showed that the 28 functional subtype B and B′ *env* clones from several HIV-1 epidemic regions of China were clearly distinguishable as B or B′-like strains. Although the B_CN sequences originated from the B_EU strains, there was no intersection between the B_CN and B_EU viruses, suggesting that genetic variation is smaller among national regions than among international regions. Therefore, analysis of the genetic and phenotypic properties of Env pseudoviruses according to their phylogenetic groups is appropriate.

The utility of any panel of Env reference strains in HIV-1 vaccine development also depends on the ability of the strains to detect known bnmAbs. In this regard, the panel selected herein was highly sensitive for detecting bnmAb epitopes in CD4bs (e.g., VRC01 and VRC03), V3 glycan (e.g., PGT121 and PGT126), and gp41 MPER (e.g., 2F5, 4E10, and 10E8). When sCD4 or some trimer apex-binding Abs were tested, the B or B′ strains from China showed features that significantly distinguished them from the standard B strains from Western countries. The unique phenotypic properties of the Chinese strains allowed vaccines targeting the epidemics in this region to be evaluated.

The amino acid diversity of the V3 loop of gp120 is reportedly related to HIV-1 preferences for coreceptor usage and disease progression^36,37^. GPGR, GPGQ, and GPGK are the most common sequences at the top of the V3 loop in subtype B^32^. In this study, the B_CN clones from the MSM cohort showed greater polymorphism in the V3 tetramer, which may reflect phenotypic differences between B_CN and B_EU. However, these genotypic differences were not obvious when the V3 glycan-targeting bnmAbs (e.g., PGT121 and PGT126) were tested against the three groups of pseudoviruses. This may be due to the high efficiency of the two antibodies targeting conserved epitopes rather than the variable sites formed by the four amino acid residues at the tip of the V3 loop.

The neutralization phenotypes were also characterized using plasma samples from HIV-1-infected individuals. The subtype-matched plasma samples showed higher neutralization potency, similar to a previous report^38^, which suggests that the neutralizing activity should be tested primarily against viruses that are matched in genetic subtype to the vaccine strain to identify the advantages of the candidates. Some pseudoviruses from Western countries presented different neutralization patterns against the HIV-1-positive plasma samples. The formerly identified tier 1 strain BZ167.12 and tier 2 strain 6535.3 acted as tier 2 and tier 1 viruses, respectively, which suggests that when a vaccine is evaluated, the viral strains should be selected with consideration of the target region.

In conclusion, we systematically analyzed and compared the characteristics of subtype B and B′ pseudoviral strains from China and a standard subtype B pseudovirus panel derived from Europe and the United States of America. Both genotypic and phenotypic differences were identified between the Chinese and European/USA B strains. These types of variabilities are major challenges in effective vaccine design and present great obstacles for vaccine-elicited response assessments. For vaccines targeting HIV-1 B and/or B′ epidemics in China, designing candidate vaccines against prevailing Chinese viruses based on *env* clones isolated from patients in China is rational. For the evaluation of NAbs elicited by vaccines, a three-tier approach is recommended to minimize the number of assays to perform in the case of weak immunogens and allows large data sets to be acquired in cases in which stronger immunogenicity is observed^12^. The molecular clones constructed in this study could be used as candidate strains to evaluate HIV-1-NAb-based candidate vaccines and to identify promising designs among these candidates in China using Chinese isolates.

## Materials and methods

### Blood samples and bnmAbs

Blood samples for cloning the *env* gene were obtained from two populations: chronically HIV-infected FPDs from different regions across China and an MSM cohort from an AIDS clinic at You’an Hospital in Beijing, China. All donors and patients were naïve to antiretroviral treatment. All samples were coded based on the region of China from which they were collected (Table 1). A plasma panel was used to test the neutralization sensitivities of all pseudoviruses, which comprised 44 HIV-1-positive plasma samples against HIV-1 subtype B/B′ (*n* = 24) against either CRF01_AE (*n* = 10) or CRF07/08_BC (*n* = 10). Written informed consent was obtained from all volunteers. All plasma samples were heat-inactivated for 1 h at 56 °C and stored at −80 °C until use.

Based on their binding sites in the *env* protein^39^, five classes of bnmAbs were used to investigate the neutralization features of the pseudoviruses. The first class of bnmAbs targeted the CD4-binding site and included IgG1b12^40^, VRC01^41^, and VRC03^41^. The second class included PG9^8^, PG16^8^, PGT145^7^, CH01^42,43^, CH02^42,43^, CH03^42,43^, and CH04^42,43^, all of which target the trimeric apex site, but with subtle differences in the exact epitopes. The third class of bnmAbs was predicted to bind the high-mannose patch on gp120 and included 2G12^44–48^. The fourth class targeting V3-glycan included PGT121^7^ and PGT126^7^. The epitopes of the fifth class, which included 2F5^44,49,50^, 4E10^51^, and 10E8^52^, were in the MPER. All bnmAbs were obtained from the National Institutes of Health AIDS Research and Reference Reagent Program (NIH ARRRP). The 2F5, 4E10, and 2G12 antibodies were donated by Dr. Hermann Katinger; PG9, PG16, PGT121, PGT126, and PGT145 were donated by IAVI; IgG1b12 was donated by Dr. Dennis Burton, and Carlos Barbas; VRC01 and VRC03 were donated by Xueling Wu, Zhi-Yong Yang, Yuxing Li, Gary Nabel, and John Mascola; CH01, CH02, CH03, and CH04 were donated by the Duke Human Vaccine Institute, Duke University Medical Center; and 10E8 was donated by Jinghe Huang, Leo Laub, and Mark Connors. The sCD4, purchased from Sino Biological Inc. (Beijing, China), was also used to test the neutralizing properties.

### Cells and plasmids

TZM-bl cells^53–57^, the *env*-deficient HIV-1 backbone (pSG3^△env^)^57,58^ and a panel of HIV-1 subtype B *env* clone^20,57,58^ plasmids were obtained from NIH ARRRP, donated by John Kappes, Xiaoyun Wu, and Tranzyme (Birmingham, AL, USA). Six tier 1 HIV-1 *env* plasmids (SF162.LS, MN.3, Bal.26, SS1196.1, BX08.16, and BZ167.12) were kindly provided by David C. Montefiori (Duke University Medical Center). Some clones (B01, B02, B03, B05, and B06) isolated in China and used in the current study were previously described by Chong et al.^22^ (Table 1).

### Construction of HIV-1 molecular clones

RNA was extracted from a 160 μl plasma sample using a QIAamp Viral RNA Kit (Qiagen GmbH, Germany) and reverse transcribed into cDNA with a SuperScript First-Strand Synthesis Kit (Invitrogen). Full-length *env* sequences were amplified with nested PCR using the primers described previously^25^. The PCR products were inserted into pCDNA3.1 after purification with the QIAquick Gel Extraction Kit (Qiagen GmbH). Competent trans5α cells (Transgen Biotech, Beijing, China) were transfected with the ligated products. Positive clones were selected and screened by restriction enzyme digestion and sequencing. The 293FT cells (Invitrogen) were cotransfected with the identified clones together with the HIV-1 backbone (pSG3^△env^). The culture supernatants were used to infect TZM-bl cells. Env clones conferring the highest infectivity were selected for further study.

### DNA sequence analysis

Sequences were aligned using the ClustalW program in MEGA 6.06 software. Phylogenetic analysis was conducted using the neighbor-joining method. Glycosylation sites were predicted with the HIV-1 database online program N-GlycoSite (http://www.hiv.lanl.gov/content/sequence/GLYCOSITE/glycosite.html).

### Pseudovirus preparation, titration, and neutralization assay

All methods were performed as described previously^16,25^.

### Data analysis

Statistical analyses of the gp160 sequence and neutralization data were performed using GraphPad Prism 6 software. The Kruskal–Wallis test was used for the neutralization potency analysis. ANOVA was used to analyze differences in the lengths of the different regions and the glycosylation sites of gp160.

The 50% inhibitory dose (ID_50_) was defined as the reciprocal of the plasma reagent dilution that caused a 50% reduction in relative luminescence units (RLU) compared with the virus control wells after subtraction of the background RLU. All ID_50_ values were calculated using an Excel macro downloaded from the HIV database (http://www.hiv.lanl.gov/content/nab-reference-strains/html/home.htm). The data validity criteria were used in accordance with the international Good Clinical Laboratory Practice laboratory standards:^21^ (1) the average RLU for virus control wells was 10 times greater than the average RLU for the cell control wells; (2) the percentage coefficient of variation (% CV) between the virus control wells was 30%; (3) the % CV of the sample wells was 30% for sample dilutions yielding at least 40% neutralization; (4) the neutralization curve was sigmoidal and approximately linear approximately 50%; and (5) the TZM-bl cells looked healthy and were not killed by the pseudovirus.

The heatmap program was used to analyze the clustering patterns for viruses and plasma pools (https://www.hiv.lanl.gov/content/sequence/HEATMAP/heatmap_mainpage.html). This strategy clusters pseudoviruses based on their susceptibility to panels of plasmas while simultaneously clustering plasmas based on their ability to neutralize a panel of pseudoviruses. The magnitude of neutralization (base-10 logarithm of ID_50_ values) is denoted by color, and the numbers in the key that corresponded to the colors are the base-10 logarithm of the ID_50_ values. A color palette was used to map the neutralization values to the colors: lower values are represented by less-saturated light colors, and higher neutralization values are represented by more-saturated dark colors.
